# Genomic characterization and preclinical evaluation of the candidate probiotic strain *Lactococcus cremoris* FBMS_5810

**DOI:** 10.3389/fmicb.2026.1812433

**Published:** 2026-04-29

**Authors:** Ioanna Farmakioti, Konstantinos Tegopoulos, Electra Stylianopoulou, Nikistratos Siskos, Lydia Angelopoulou, Andreas Rafail Vasileiou, Evangelia Karagianni, Dionysios Kandylas, Fragkiski Fragkiskatou, Chrysoula Pavlatou, Alexandra Tsaroucha, Petros Ypsilantis, Yiannis Kourkoutas, Petros Kolovos, George Skavdis, Maria E. Grigoriou

**Affiliations:** 1 Department of Molecular Biology and Genetics, Democritus University of Thrace, Alexandroupolis, Greece; 2 Department of Medicine, Democritus University of Thrace, Alexandroupolis, Greece

**Keywords:** *Lactococcus cremoris* FBMS_5810, microbiome, preclinical study, probiotics, whole-genome sequencing

## Abstract

This study presents a comprehensive genomic and preclinical evaluation of *Lactococcus cremoris* FBMS_5810, establishing its taxonomic identity, genomic uniqueness, and safety profile. Genomic analyses identified strain-specific genes linked to adhesion, colonization, and pathogen exclusion, aligning with previously observed *in vitro* probiotic properties. *In vivo* studies in healthy mice demonstrated that *Lactococcus cremoris* FBMS_5810 modulates gut microbiota composition. Specifically, the relative abundance of Muribaculaceae, Erysipelotrichaceae, and Streptococcaceae was significantly increased in the probiotic-treated group, whereas the relative abundance of Ruminococcaceae, Bacteroidaceae, Porphyromonadaceae, and Dehalobacteriaceae was decreased. Administration of *Lactococcus cremoris* FBMS_5810 was also associated with changes in intestinal gene expression: in the ileum, *Tnf* and *Il1b* expression increased, while in the cecum, *Zo1* expression was elevated. These findings may indicate a role in supporting intestinal homeostasis and could be linked to reduced susceptibility to diet- and inflammation-related disorders. Overall, these results suggest that *Lactococcus cremoris* FBMS_5810 may be a useful candidate for further investigation in the development of health-oriented microbial products. By integrating genomic characterization with preclinical evaluation, this study not only highlights *Lactococcus cremoris* FBMS_5810 as a promising candidate but also provides a systematic approach for the identification and validation of probiotics, advancing both fundamental understanding and translational applications in molecular microbiology.

## Introduction

Microorganisms inhabit the human and animal body in a complex relationship, with approximately 95% of them residing in the gastrointestinal (GI) tract—predominantly within the large intestine; the stomach and small intestine remain less densely colonized due to their inhospitable environment (Hou et al., 2022; Lkhagva et al., 2021; Maftei et al., 2024; de Vos et al., 2022). Even though the physiology of the GI tract is well understood, the trillions of microorganisms—including bacteria, viruses, fungi, and protozoa—that inhabit the gut, collectively known as the gut microbiota, have emerged as a major focus of research over the past two decades. The term “human microbiome” was introduced by Joshua Lederberg, who defined it as “the ecological community of commensal, symbiotic, and pathogenic microorganisms that literally share our body space and have been all but ignored as determinants of health and disease” (Lederberg and McCray, 2001). In this broad sense, the gut microbiome refers not only to the microbial community itself, but also to its interactions with the host. This complex system influences numerous aspects of health, including energy and nutrient extraction, biosynthesis of bioactive molecules, and maintenance of epithelial barrier integrity (Gomaa, 2020; Jandhyala et al., 2015; Kho and Lal, 2018). Additionally, the gut microbiome plays a central role in defending against pathogens and in shaping and modulating both the immune and nervous systems (Gomaa, 2020; Jandhyala et al., 2015; Kho and Lal, 2018).

Colonization of the human gut begins *in utero* and undergoes dynamic changes during the first 2 years of life, eventually stabilizing into a complex community dominated by Firmicutes and Bacteroidetes (Maftei et al., 2024). However, multiple, early-life factors—such as mode of delivery, feeding practices, antibiotic exposure, environmental influences and diet—affect both early establishment, as well as lifelong composition and diversity of the gut microbiota, resulting in each individual developing a unique “gut microbiota fingerprint” (Pedroza Matute and Iyavoo, 2023; Wen and Duffy, 2017). While a universally accepted definition of a “normal” or “healthy” gut microbiota is still lacking (Piquer-Esteban et al., 2021), specific alterations in microbial community structure known as dysbiosis have been linked with gastrointestinal, metabolic, cardiovascular, neurologic, and immune-related disorders (Chen et al., 2021; Gomaa, 2020; Khalil et al., 2024; Kho and Lal, 2018).

Probiotics are defined by the Food and Agriculture Organization of the United Nations (FAO) and the World Health Organization (WHO) as “live microorganisms which, when administered in adequate amounts, confer a health benefit on the host” (Hill et al., 2014). Although the molecular mechanisms underlying probiotic-host interactions remain largely unknown, a substantial body of evidence suggests that administration of probiotic strains can help alleviate symptoms in conditions such as inflammatory bowel disease (IBD), obesity, diabetes, diarrhea, non-alcoholic fatty liver disease (NAFLD), hypertension, atherosclerosis, allergies and cancer (Gul and Durante-Mangoni, 2024; Maftei et al., 2024; Petrariu et al., 2024). These effects appear to be mediated through several mechanisms, including metabolic regulation, vitamin synthesis, suppression of pathogens, strengthening of the intestinal epithelial barrier and immunomodulation (Maftei et al., 2024; Markowiak and Śliżewska, 2017).

Despite the availability of diverse probiotic products in the market, a central question still persists: do they offer benefits only for individuals with pathological conditions, or can they also support general health in otherwise healthy populations? Current evidence remains inconclusive regarding the benefits of probiotic administration for healthy individuals (Khalesi et al., 2019; Merenstein et al., 2024). Moreover, a systematic review and meta-analysis showed that the probiotic effects cannot be generalized even across different strains within the same species, underscoring the importance of strain specificity (McFarland et al., 2018); this has prompted growing interest in the isolation and characterization of novel probiotic strains with defined functions.

The advances in high-throughput sequencing technologies have significantly deepened our understanding of the intestinal microbiome, which harbors over 3 million genes—far exceeding the ∼23,000 genes in the human genome—and produces a wide variety of bioactive metabolites (Qin et al., 2010). In addition, whole-genome sequencing (WGS) and genomic characterization have been essential for accurate taxonomic identification and evaluation of properties relevant to health and food applications. Regulatory bodies, such as the European Food Safety Authority (EFSA), mandate WGS and genome annotation for strains intended for biotechnological or health-related applications (EFSA Panel on Additives and Products or Substances used in Animal Feed [FEEDAP], 2018). Consequently, candidate probiotic strains are increasingly evaluated using complementary approaches that combine genomic, *in vitro*, and *in vivo* analyses to establish both safety and functionality (López-Palestino et al., 2025).

In our previous study, we isolated the strain *Lactococcus cremoris* FBMS_5810 and evaluated a set of probiotic features *in vitro* (Pavlatou et al., 2025). Building upon these findings, the present study provides a more comprehensive characterization of the strain. Specifically, we combine whole-genome analysis to assess novelty, safety, and phylogenetic placement, with preclinical testing in a murine model to evaluate the physiological effects of *Lactococcus cremoris* FBMS_5810 administration. Together, our results provide a basis for considering *Lactococcus cremoris* FBMS_5810 as a promising probiotic candidate for future applications.

## Materials and methods

### Bacterial culture conditions

*L. cremoris* FBMS_5810 was cultured under anaerobic conditions on a sterile food-grade medium (20 g/L glucose, 25 g/L yeast extract, 2 g/L KH_2_PO_4_, 6 g/L CH_3_COONa, 0.3 g/L MgSO_4_, and 0.005 g/L MnSO_4_) at 30 °C for 24 h. Freeze-dried cells were obtained following established protocols (Prapa et al., 2025), and their viability throughout storage was verified through microbiological assays.

### Bacterial identification and classification

#### Genomic DNA extraction and whole genome sequencing

*L. cremoris* FBMS_5810 cells were harvested by centrifugation of 3 mL of overnight bacterial culture at 5,000 × g for 10 min at room temperature. The supernatant was discarded, and total genomic DNA was extracted from the pellet using the NucleoSpin Stool Mini Kit (Macherey-Nagel, Düren, Germany), following the manufacturer’s protocol. For DNA quantitation, the Qubit dsDNA HS Assay Kit (Thermo Fisher Scientific, Waltham, MA, United States) was used and measurements were taken in the Qubit 4 Fluorometer (Thermo Fisher Scientific, Waltham, MA, United States). DNA integrity was evaluated by electrophoresis on a 1% w/v agarose (UltraPure Agarose, Invitrogen, Carlsbad, CA, United States) gel pre-stained with GelRed Nucleic Acid Gel Stain (Biotium, Fremont, CA, United States).

Enzymatic fragmentation of 400 ng of genomic DNA into blunt-ended fragments, with an average size of 300 bp, was performed using the Ion Xpress™ Plus Fragment Library Kit (Thermo Fisher Scientific, Waltham, MA, United States), following the manufacturer’s protocol. Fragmented DNA was purified using NucleoMag NGS Clean-up and Size Select magnetic beads (Macherey-Nagel, Düren, Germany) at a 1/1.8 (DNA/beads) volume ratio. The desired DNA fragment size was confirmed by electrophoresis on a 2% w/v agarose (UltraPure Agarose, Invitrogen, Carlsbad, CA, United States) gel pre-stained with GelRed Nucleic Acid Gel Stain (Biotium, Fremont, CA, United States). The purified fragmented DNA was then used for library construction with the Ion Plus Fragment Library Kit (Thermo Fisher Scientific, Waltham, MA, United States), as per the manufacturer’s instructions. The DNA of the library underwent an additional purification step using magnetic beads at a 1/1.2 (DNA/beads) ratio and was subsequently quantified by quantitative Polymerase Chain Reaction (qPCR) using the Ion Universal Library Quantitation Kit (Thermo Fisher Scientific, Waltham, MA, United States) on a StepOne™ Real-Time PCR System (Applied Biosystems, Foster City, CA, United States). Finally, 100 pM of the library was used for template preparation on the Ion Chef™ System (Thermo Fisher Scientific, Waltham, MA, United States), and sequencing was conducted on the Ion Torrent GeneStudio™ S5 System (Thermo Fisher Scientific, Waltham, MA, United States) at the Department of Molecular Biology & Genetics, according to the manufacturer’s instructions.

#### Genome annotation and phylogenetic analysis

Raw reads of *L. cremoris* FBMS_5810 obtained from the Ion Torrent S5 platform were filtered to retain sequences with lengths between 70 and 370 bp and a minimum Phred quality score of Q20. Filtering and quality control were performed using samtools (version 1.17) (Danecek et al., 2021), fastq_filter (version 0.3.0X.X) (LUMC, 2025), and seqtk (version 1.5) (lh3, 2025). Genome assembly construction was carried out using SPAdes (version 4.2.0) (Prjibelski et al., 2020), and assembly statistics were calculated with QUAST (version 5.2.0) (Gurevich et al., 2013). Annotation was conducted using the Prokaryotic Genome Annotation Pipeline (PGAP) (Tatusova et al., 2016), which also screened for contamination and generated GenBank-compliant files. The circular genome map was created using CGView (Stothard and Wishart, 2005).

Species identification was performed using PGAP taxonomic assignment and Kraken2 (version 2.1.3) (Wood et al., 2019, p. 2). To confirm the novelty of the strain, comparative genomic analyses were conducted. FastANI (v1.33) (Jain et al., 2018) was utilized to calculate average nucleotide identity (ANI) and Roary (version 3.13.0) (Page et al., 2015) for pangenome analysis, incorporating all publicly available *Lactococcus cremoris* genomes from GenBank (as of June 2025). Phylogenetic trees generated by Roary were visualized with Itol (Letunic and Bork, 2016).

Functional classification of predicted proteins was performed using eggNOG-mapper (Cantalapiedra et al., 2021), assigning them to Clusters of Orthologous Groups (COG) functional categories. Subcellular localization predictions were conducted using PSORTb (version 3.0) (Yu et al., 2010) to classify proteins into cytoplasmic, membrane-associated, cell wall, or extracellular localizations. Putative prophage sequences were detected with VirSorter2 (v2.2.4) (Guo et al., 2021), and antibiotic resistance genes were identified using the Resistance Gene Identifier (RGI, version 6.0.3) (Alcock et al., 2023) against the Comprehensive Antibiotic Resistance Database (CARD) (Alcock et al., 2023). The potential pathogenicity of the strain was examined using PathogenFinder2 (Florensa et al., 2025) and VirulenceFinder-2.0 (Joensen et al., 2014). Finally, stress response genes were identified using in-house scripts.

### Animals and experimental design

Animal experiments were carried out following the guidelines established by the European Union. The protocol was approved by the committee for the Care and Use of Laboratory animals of our Institution and of the Prefecture of Eastern Macedonia and Thrace, Greece, under permit number 36662/118 (08/02/2022).

C57BL/6J male mice, 12–13 weeks old, were housed in individually ventilated cages (IVCs) in the Laboratory of Experimental Surgery and Surgical Research of the Department of Medicine of the Democritus University of Thrace, under controlled conditions (12 h light/dark cycle with 21 ± 2 °C and 55 ± 10% relative humidity) and with *ad libitum* access to sterile laboratory chow (Mucedola, Italy, type 4RF25) and water.

Two experimental groups were considered: the PBS group, involving mice receiving sterile phosphate-buffered saline (PBS, Invitrogen, Waltham, MA, United States), and the LC group, involving mice receiving *L. cremoris* FBMS_5810 cells resuspended in PBS (see below). After G*Power 3.1 analysis (Faul et al., 2009), the required sample size was determined to six animals per group (*n* = 6). Mice were randomly divided into the two groups and maintained in separate cages according to their experimental group. During group allocation, potential confounding factors were controlled to achieve balanced group composition. Specifically, sibling animals from the same litter were distributed across the experimental groups to minimize litter effects. In addition, body weight was recorded prior to the intervention and animals were assigned to ensure comparable weight distribution across groups.

After a 7-day acclimatization period, the PBS group received 0.2 mL of sterile PBS daily from days 1 to 7, and every other day for the subsequent 35 days; the LC group was administered 10∧9 freeze-dried bacterial cells of *L. cremoris* FBMS_5810 resuspended in 0.2 mL of sterile PBS, following the same dosing regimen. The administration was performed by trained personnel via oral gavage using appropriate gavage tubes to minimize animal stress. Mice were closely monitored throughout the intervention period to ensure their well-being and check for potential discomfort. Body weight was recorded on a weekly basis. Before the first gavage (Day 0), as well as 1 day after the last gavage, fecal samples were collected using metabolic cages to prevent their contamination with urine, instantly frozen in dry ice and stored immediately at −80 °C for subsequent microbiome analysis. At the end of the intervention, mice were euthanized via cervical dislocation. Immediately after confirming death by the absence of the toe pinch reflex, blood was collected in K2EDTA-containing tubes (Sarstedt, Germany) via cardiac puncture (closed technique), following the NC3Rs guidelines (accessed: 30 Nov 23).^1^ Serum levels of blood urea nitrogen (BUN), creatinine (CREA), high-density lipoprotein (HDL), low-density lipoprotein (LDL), total cholesterol (TC), and triglycerides (TG), were analyzed at an accredited diagnostic company (LABnet, Thessaloniki, Greece). Intestinal (colon, cecum, and ileum) tissues were extracted and rinsed in cold PBS to eliminate residual intestinal contents. Half of each tissue was fixed in 4% w/v paraformaldehyde (PFA) in PBS at 4 °C for hematoxylin/eosin staining, and the other half was instantly frozen in dry ice and stored at −80 °C for subsequent gene expression analysis.

### Histological analysis

Fixed tissue samples were rinsed in PBS and then cryoprotected by immersion in 30% w/v sucrose in PBS at 4 °C. Following this, they were embedded in Tissue Freezing Medium (Leica, Germany), cut into 12-μm-thick sections using a Leica CM1900 UV cryostat (Leica, Germany) and loaded onto plain glass slides. Tissue sections were stained with hematoxylin and eosin (Applichem, Darmstadt, Germany) for morphology observation under a Leica DM5500B microscope (Leica Microsystems, Germany) equipped with a DFC7000T digital camera (Leica Microsystems, Germany). Digital images were acquired using the LAS v4.13 imaging software (Leica Microsystems, Germany), while figure layouts were created using the GIMP (version 2.10.38,^2^ accessed on 16 July 2025). Crypt depth and villus height were measured in ImageJ (version 2.9.0/1.53t,^3^ accessed on 10 July 2025).

### Analysis of fecal microbiome

#### Fecal DNA extraction and 16S rRNA sequencing

Metagenomic DNA was isolated from fecal samples with the NucleoSpin Stool Mini Kit (Macherey-Nagel, Düren, Germany), according to the manufacturer’s instructions. The concentration and purity of the isolated DNA were determined spectrophotometrically at 260 nm using the NanoDrop Spectrophotometer (Thermo Fisher Scientific, Waltham, MA, United States), while DNA integrity was verified via electrophoresis on a 1% w/v agarose (UltraPure Agarose, Invitrogen, Carlsbad, CA, United States) gel pre-stained with GelRed Nucleic Acid Gel Stain (Biotium, Fremont, CA, United States).

The V4 region of the 16S rRNA gene was amplified from the fecal metagenomic DNA with the primers 5’-GTGCCAGCMGCCGCGGTAA-3’ (forward) and 5’-GGACTACHVGGGTWTCTAAT-3’ (reverse), generating a 290 bp amplicon. PCR was carried out in a VeritiPro Thermal Cycler (Thermo Fisher Scientific, Waltham, MA, United States) using KAPA SYBR FAST qPCR Master Mix (2 × ) (Sigma-Aldrich, St. Louis, MO, United States) under the following conditions; 95 °C for 5 min, 25 cycles of 95 °C for 30 s, 58 °C for 40 s, 72 °C for 40 s, and a final elongation step at 72 °C for 5 min. PCR products were subjected to electrophoresis on a 2% w/v agarose (UltraPure Agarose, Invitrogen, Carlsbad, CA, United States) gel pre-stained with GelRed Nucleic Acid Gel Stain (Biotium, Fremont, CA, United States) to confirm successful amplification, and then were purified with NucleoMag NGS Clean-up and Size Select magnetic beads (Macherey Nagel, Düren, Germany), at a 1/1.8 (DNA/beads) volume ratio. The concentration of the purified PCR products was evaluated using Qubit 4 Fluorometer (Thermo Fisher Scientific, Waltham, MA, United States) with the Qubit dsDNA HS Assay Kit (Thermo Fisher Scientific, Waltham, MA, United States). Library construction and sequencing were carried out on the Ion Torrent S5 platform (Thermo Fisher Scientific, Waltham, MA, United States) as previously described (Tegopoulos et al., 2024).

#### Sequencing data analysis

The Torrent Suite software (Thermo Fisher Scientific, Waltham, MA) provides raw sequencing data, which has been processed to eliminate polyclonal, low-quality and low-signal reads. The obtained UBAM files were converted to fasta format files with Samtools (version 1.13) (Li et al., 2009) and then analyzed with Mothur (version 1.45.3) (Schloss et al., 2009), as previously described (Tegopoulos et al., 2024). A 0.1% abundance filter combined with a 25% prevalence rule was applied to filter out low-abundance bacterial OTUs using the Microbiome Analyst online platform,^4^ accessed on 6 July 2025) (Lu et al., 2023). Diversity indices, Principal Coordinates Analysis (PCoA) based on a Bray-Curtis dissimilarity matrix, and Permutational Multivariate Analysis of Variance (PERMANOVA) were conducted using the PAST (PAleontological STatistics) software package (version 4.03) (Hammer et al., 2001), while relative abundance graphs were generated using GraphPad Prism 9.0 (GraphPad Software, CA, United States).

### Gene expression analysis

RNA from the intestinal tissues was extracted using Trizol Reagent (Thermo Fisher Scientific, Waltham, MA, United States), according to the manufacturer’s protocol. The quantity and purity of the isolated RNA were evaluated using a NanoDrop Spectrophotometer (Thermo Fisher Scientific, Waltham, MA, United States). RNA integrity was assessed by electrophoresis of 100 ng of total RNA on a 1.5% w/v agarose (UltraPure Agarose, Invitrogen, Carlsbad, CA, United States) gel pre-stained with GelRed Nucleic Acid Gel Stain (Biotium, Fremont, CA, United States). RNA was reverse transcribed with the PrimeScript RT Reagent Kit (Takara Biotechnology, Dalian, China), following the manufacturer’s instructions. cDNA was stored at −20°C for future use. qPCR was performed as previously described (Farmakioti et al., 2025). Raw data generated from StepOneTM Software (version 2.3) were processed using the LinRegPCR program (version 2021.2) (Ramakers et al., 2003) to perform baseline correction and calculate PCR efficiency for each reaction. Fold changes in the expression of the target genes were assessed using the common base method as described by Ganger et al. (2017), with *Canx* serving as the reference gene and the PBS group as the reference condition.

### Statistical analysis

GraphPad Prism 9.0 software (GraphPad Software, CA, United States) was used for statistical analysis. Depending on the data distribution, the statistical significance was evaluated using either unpaired *t*-test or unpaired Mann–Whitney U-test, both with two-tailed distributions. *p*-value < 0.05 was considered as statistically significant. Effect sizes were subsequently estimated using the JASP software package (version 0.19.3). They were expressed as Cohen’s d (d) for the *t*-test and as the rank biserial correlation (r) for the Mann–Whitney U-test. Effect sizes were interpreted according to conventional thresholds: small (d: 0.20–0.50; r: 0.10–0.30), intermediate (d: 0.50–0.80; r: 0.30–0.50), and large (d ≥ 0.80; r: 0.50–1.00). The sign of the effect size indicates the direction of the observed effect.

## Results

### Genome sequencing, assembly, and annotation

**FIGURE 1:**
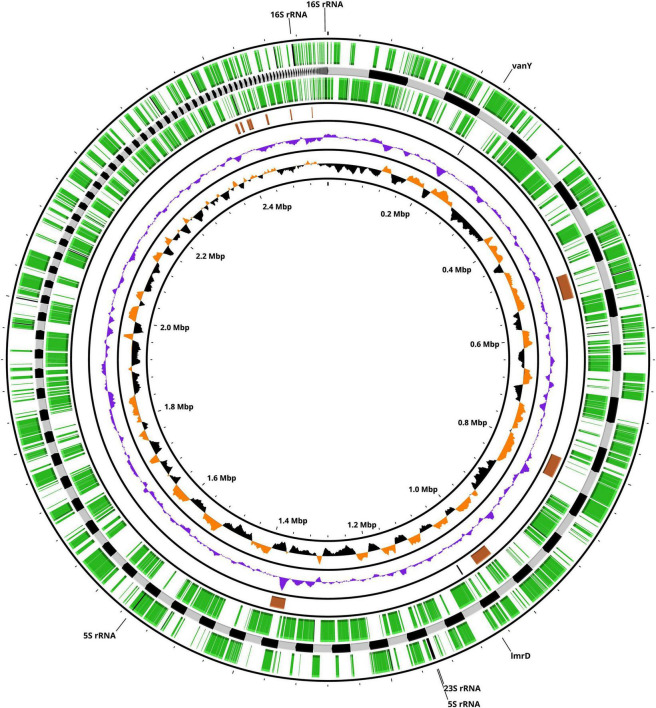
Circular chromosomal map of *Lactococcus cremoris* FBMS_5810, highlighting genomic organization, compositional features, and the distribution of prophage regions and antibiotic resistance–related genes. Concentric rings represent genomic features arranged from the center outward as follows: (1) genome size scale in megabase pairs (Mbp); (2) GC skew with positive values shown in orange and negative values in black; (3) GC content (purple); (4) prophage regions (brown) and antibiotic resistance-related genes (black); (5) coding sequences (CDS) on the reverse strand (green); (6) contig boundaries indicated by alternating black and grey segments; (7) coding sequences (CDS) on the forward strand (green). Outside the circular map, (8) labels indicate predicted antibiotic resistance genes and ribosomal RNA (rRNA) genes (5S, 16S, and 23S).

A total of 5,639,992 high-quality reads were obtained for the genome of *Lactococcus cremoris* FBMS_5810, corresponding, after quality filtering, to 797,008,776 bases. Genome assembly resulted in 224 contigs ≥ 200 bp, with a GC content of 35.46% and a total length of 2,559,729 bp, yielding an estimated coverage of approximately 312 × . Annotation using the NCBI Prokaryotic Genome Annotation Pipeline (PGAP) identified 2,618 genes, including 2,568 coding sequences (CDSs), 50 RNA genes (comprising 2 5S rRNAs, 2 16S rRNAs, 1 23S rRNA, 42 tRNAs, and 3 ncRNAs), as well as 92 pseudogenes. A circular chromosomal representation of the genome, including annotated coding sequences, GC skew, antibiotic resistance genes, and prophage regions, is shown in Figure 1.

### Taxonomic placement, genomic comparison, and novelty confirmation

Both PGAP and Kraken2 confidently identified strain FBMS_5810 as *L. cremoris*, with PGAP reporting a high-confidence match to *L. cremoris* species (NCBI taxID = 1,359). To further investigate its correct taxonomic placement and assess potential genomic novelty, we curated a high-confidence dataset of *L. cremoris* genomes. Starting from all available assemblies labeled as *L. cremoris* in GenBank (*n* = 222, as of June 2025), we applied a series of stringent quality control and dereplication steps. A total of 25 assemblies belonging to *L. cremoris* subsp. cremoris A76 (NCBI taxID: 1104322) were removed due to poor assembly quality, as they were labeled as “contaminated, genome length too small” in the NCBI genome database; they originated from metagenomic data. However, the type strain of the subspecies (*Lactococcus cremoris* subsp. cremoris A76—GCA_000236475.1) met assembly quality criteria and was kept for the downstream analysis. Four assemblies (GCA_000447845.1, GCA_036452445.1, GCA_036452495.1 and GCA_000447825.1) were excluded due to unusually small genome sizes, while nine assemblies (GCA_009661855.1, GCA_009661865.1, GCA_009661875.1, GCA_009661885.1, GCA_009661935.1, GCA_009661955.1, GCA_009661985.1, GCA_001622305.1, and GCA_958441155.1) were removed from the analysis because their status in the NCBI database has been updated to “suppressed” due to taxonomic anomalies. To minimize redundancy within the dataset, we identified and excluded replicate genomes based on pairwise ANI > 99.9% and bidirectional coverage > 99%, retaining only one representative genome per group, thus we retained only the first genome from the replicate sets: GCA_036670545.1, GCA_036670705.1, and GCA_036670615.1; GCA_027707025.1 and GCA_027707045.1; and GCA_002078375.4 and GCA_016921055.1. Following this curation process, a total of 179 high-quality, non-redundant *L. cremoris* genomes were included in the comparative dataset that we used.

**FIGURE 2:**
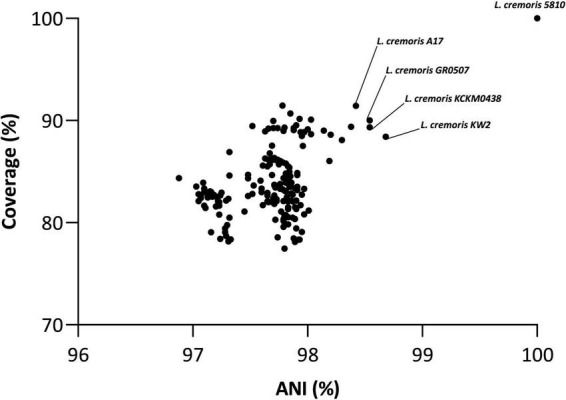
Average Nucleotide Identity and alignment coverage values between *Lactococcus cremoris* FBMS_5810 and 179 publicly available *L. cremoris* genomes showing consistently high ANI values (>95%) that confirm species-level assignment and variable alignment coverage reflecting differences in genome content among strains. Each point represents a pairwise comparison between *L. cremoris* FBMS_5810 and a single genome, with ANI (%) on the x-axis and alignment coverage (%) on the y-axis. The self-comparison of strain FBMS_5810 (100% ANI and 100% coverage) is shown in green. The closest relative, *L. cremoris* KW2, is highlighted in red, while the next two closest strains based on ANI (*L. cremoris* GR0507 and *L. cremoris* KCKM0438) are indicated in yellow.

The ANI analysis together with sequence comparison of the gapA gene (encoding glyceraldehyde-3-phosphate dehydrogenase)—previously reported as a reliable phylogenetic marker for distinguishing *Lactococcus* species (Willemoës et al., 2002)—both supported the assignment of strain FBMS_5810 to the *L. cremoris* species. Strain FBMS_5810 exhibited ANI values above 95% with all curated genomes (Figure 2 and Supplementary Table S1), confirming its species-level classification. However, ANI values remained below the 99% threshold across all comparisons, indicating genomic divergence from currently available *L. cremoris* strains. The closest relative was *L. cremoris* subsp. *cremoris* KW2 (98.68%) followed by *L. cremoris* GR0507 (98.54%) and *L. cremoris* KCKM 0438 (98.54%) (Figure 2).

**FIGURE 3:**
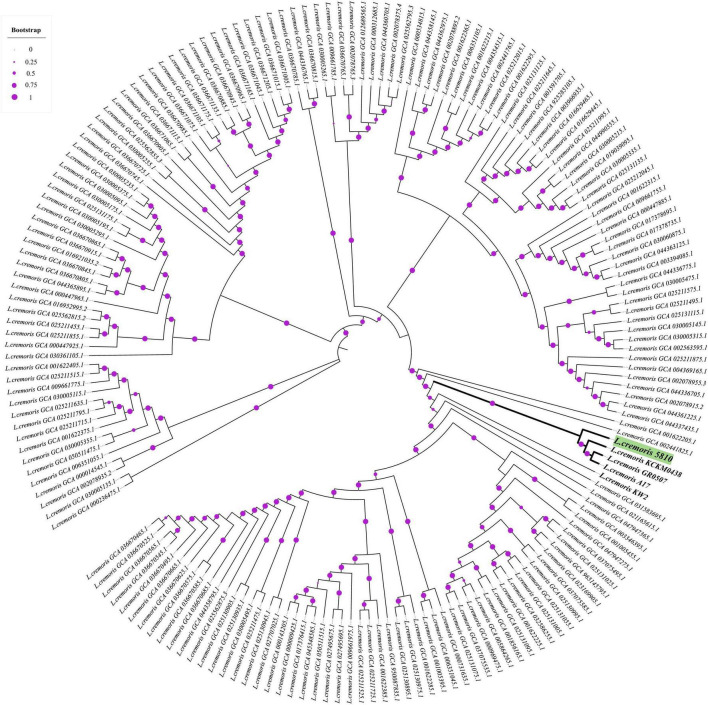
Phylogenetic tree based on accessory gene presence–absence across 180 *L. cremoris* genomes, showing that FBMS_5810 clusters with its closest relatives with strong statistical support. Bootstrap support values are represented as purple circles at each node, with circle size proportional to confidence levels. *L. cremoris* FBMS_5810 is highlighted in green and groups with its closest related strains (shown in bold) with high confidence (bootstrap = 0.99).

Pangenome analysis using Roary across a curated dataset of 180 *Lactococcus cremoris* genomes (including the genomes of the strain 5,810 and the 179 high-quality, non-redundant publicly available *L. cremoris* genomes) identified a total of 18,809 gene clusters. Among these, 770 genes were conserved across ≥ 99% of strains, constituting the core genome, while 258 genes formed the soft core (present in 95–98% of genomes). The accessory genome comprised 2,648 shell genes (15–94%) and 15,133 cloud genes (≤14%), reflecting the substantial genomic diversity within the species. *L. cremoris* FBMS_5810 harbored 157 strain-specific genes not detected in any of the other 179 genomes, supporting its designation as a genomically distinct member of the *L. cremoris* species. To further explore the phylogenetic position of strain FBMS_5810, a maximum-likelihood tree was constructed (Figure 3) based on the accessory genome (Roary output), revealing that *L. cremoris* FBMS_5810 clusters closely with *L. cremoris* KCKM0438, *L. cremoris* GR0507, *L. cremoris* A17, and *L. cremoris* KW2 —consistent with the ANI-based findings (Figure 2).

Overall, the ANI values, phylogenetic placement, and pangenome characteristics consistently support the designation of strain *L. cremoris* FBMS_5810 as a novel and genomically distinct representative within *L. cremoris* species.

### Functional annotation, subcellular localization and safety inspection

**FIGURE 4:**
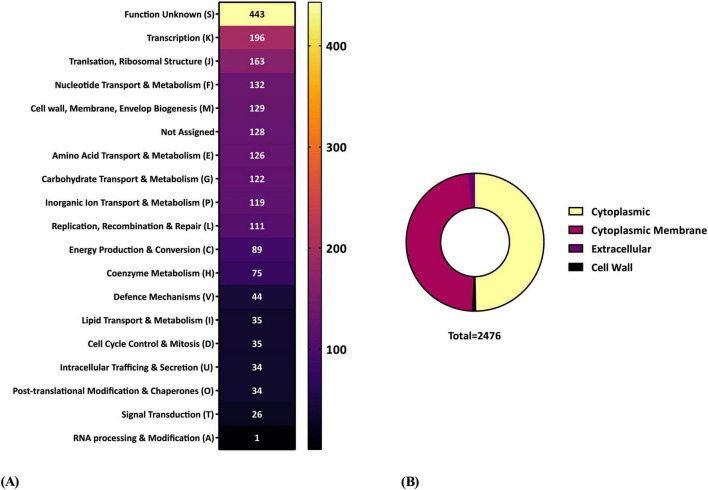
Functional categorization and subcellular localization of the predicted proteome of *Lactococcus cremoris* FBMS_5810, showing a broad distribution of proteins across COG functional categories and a predominance of cytoplasmic proteins. **(A)** Distribution of predicted proteins across the 18 COG functional categories witheach cell indicating the number of proteins assigned to a given category. **(B)** Subcellular localization of the predicted proteome based on PSORTb analysis with the pie-chart showing the proportion of proteins predicted to be cytoplasmic, cytoplasmic membrane–associated, cell wall- associated, or secreted extracellularly.

Following genome characterization and taxonomic assessment, we sought to explore the functional landscape of the predicted proteome. A Clusters of Orthologous Groups (COG)-based annotation was performed to classify proteins into broad functional categories, while subcellular localization predictions provided insights into their potential roles and functions. Out of 2,476 proteins, 2,143 were assigned to a total of 18 COG categories. The most abundant was “Function unknown” (S, 443 proteins), followed by “Transcription” (K, 196), “Translation, ribosomal structure and biogenesis” (J, 163), “Nucleotide transport and metabolism” (F, 132), and “Cell wall/membrane/envelope biogenesis” (M, 129) (Figure 4). To complement the functional annotation, we also investigated the spatial distribution of the proteome within the cell. Subcellular localization analysis indicated that 1,231 proteins were cytoplasmic, 1,179 associated with the cytoplasmic membrane, 25 localized to the cell wall, and 41 were predicted to be extracellular (Figure 4).

The genome of *L. cremoris* FBMS_5810 was then assessed for genomic features related to safety. Screening for antibiotic resistance genomic elements against the Comprehensive Antibiotic Resistance Database (CARD) identified two resistance-associated genes: a glycopeptide resistance-related gene (vanY) located within a putative vanG cluster, and the multidrug efflux transporter lmrD, associated with lincosamide resistance via an ATP-binding cassette (ABC) efflux mechanism. Notably, vanY displayed only 35.4% sequence identity compared to reference, suggesting limited functionality or a divergent variant. In contrast, lmrD showed 99.3% identity and full-length coverage; however it encodes an intrinsic efflux pump commonly found in *Lactococcus* species, which is not typically regarded as a probiotic safety concern (Lubelski et al., 2007). In terms of pathogenicity, *L. cremoris* FBMS_5810 was classified as “Human Non-Pathogenic” by PathogenFinder2, while no virulence factors were detected by VirulenceFinder-2.0. Additionally, prophage prediction revealed 11 prophage-like regions, sequences derived from 7 dsDNA phages and 4 ssDNA phages, ranging in size from 847 to 40,459 bp.

### Predicted antimicrobial traits

Concerning the antimicrobial profile of *Lactococcus cremoris* FBMS_5810, BAGEL4 identified two putative class II bacteriocin operons corresponding to lactococcin A and garvicin Q, both commonly found in *Lactococcus* strains (Tang and Leisner, 2025). The lactococcin A operon comprises four genes: a structural bacteriocin (LCR5810_000042), a bacteriocin immunity protein (LCR5810_000041), a transport-associated protein (LCR5810_000045) and a transcriptional regulator (LCR5810_000043). The garvicin Q-like operon is similarly organized, with genes encoding the bacteriocin (LCR5810_001239), its immunity protein (LCR5810_001240), an MFS-type transporter protein (LCR5810_001241), and a transcriptional regulator (LCR5810_001242).

Further *in silico* analyses of the two identified bacteriocins revealed inconsistencies in their predicted secretion and localization. Lactococcin A harbors a high-confidence Sec/SPI signal peptide, indicative of secretion via the general Sec pathway, yet PSORTb predicted its localization as cytoplasmic. In contrast, garvicin Q lacks a canonical signal peptide, but it was predicted to be extracellular, potentially due to the presence of a proximal MFS-type transporter that may mediate non-classical export, as previously proposed (Soltani et al., 2021). These conflicting *in silico* predictions highlight the need for *in vitro* validation of the expression and functionality of the above-mentioned putative bacteriocin clusters.

### Stress adaptation

In order to investigate the organism’s potential to withstand the diverse and often harsh conditions of the gastrointestinal tract, we scanned its genome to find putative genes/proteins associated with stress response. Notably, *L. cremoris* FBMS_5810 encodes a broad repertoire of previously described thermal and cold stress response genes (Mileriene et al., 2023). Thermal stress adaptation is supported by the presence of several conserved heat shock proteins, including DnaJ (LCR5810_001859), DnaK (LCR5810_001968), Hsp33 (LCR5810_000664), GrpE (LCR5810_001967), and the GroEL/GroES chaperonin system (LCR5810_002434, LCR5810_002433). Moreover, a cold shock protein CspA (LCR5810_000760) was also identified.

Furthermore, the strain’s ability to cope with acid stress was assessed by genomic analysis, which revealed the presence of genes encoding key components of the ATP synthase complex (subunits A, B, and C; LCR5810_001700, LCR5810_001699, LCR5810_001701), which help regulate intracellular pH through proton extrusion. In addition, the detection of glucose-6-phosphate isomerase (LCR5810_002288) and pyruvate kinase (LCR5810_002106) suggests glycolytic activity that may support energy production and acid resistance under stress conditions. The genome also harbors an alkaline shock response membrane anchor protein AmaP (LCR5810_002220), which may contribute to adaptation in alkaline environments, such as during passage through the intestine or under food processing conditions.

### Surface-associated proteins and host interaction

Among the 25 proteins predicted to localize the cell wall, 17 contained LPxTG motifs, highlighting their potential for covalent anchoring to the peptidoglycan layer—a classical mechanism by which Gram-positive bacteria attach surface proteins to the cell envelope. Several of these LPxTG-anchored proteins also harbor known adhesion-associated domains, including mucin-binding domains (MucBP), pilin-related domains (e.g., SpaA/SpaH, pilin N-terminal), and collagen-binding motifs (Cna B-type), suggesting strong potential for mucosal adherence and host interaction. A detailed list of the predicted cell wall proteins, their localization motifs, and the presence of signal peptides is provided in Table 1.

**TABLE 1:** *In silico*—identified cell wall–localized proteins in *L. cremoris* FBMS_5810 with conserved motifs and signal peptides.

No.	Gene ID	Motif within protein	Signal peptide
1	LCR5810_001476	LPXTG cell wall anchor/Mucin-binding domain	No
2	LCR5810_000498	LPXTG cell wall anchor	Yes
3	LCR5810_000554	Collagen-binding domain	Yes
4	LCR5810_000934	LPXTG cell wall anchor	No
5	LCR5810_000935	LPXTG cell wall anchor/Pilin-related domain	Yes
6	LCR5810_000937	LPXTG cell wall anchor/Pilin-related domain	No
7	LCR5810_001400	LPXTG cell wall anchor	No
8	LCR5810_001605	LPXTG cell wall anchor	Yes
9	LCR5810_002114	Adhesive domain	Yes
10	LCR5810_002211	LPXTG cell wall anchor/Mucin-binding domain	Yes
11	LCR5810_002294	LPXTG cell wall anchor	No
12	LCR5810_002376	LPXTG cell wall anchor/Pilin-related domain	Yes
13	LCR5810_002377	LPXTG cell wall anchor/Pilin-related domain	No
14	LCR5810_000232	LPXTG cell wall anchor	Yes
15	LCR5810_000477	LPXTG cell wall anchor/Pilin-related domain	No
16	LCR5810_000486	LPXTG cell wall anchor	No
17	LCR5810_000573	LPXTG cell wall anchor	No

In addition to the cell wall-localized proteins, 41 proteins were predicted to be extracellular, 13 of which carry a predicted signal peptide, supporting the notion of active secretion. This localization is often associated with secreted enzymes, signaling molecules, and antimicrobial factors. Notably, several extracellular proteins contained conserved domains, such as LysM (peptidoglycan-binding) (Buist et al., 2008), WxL (non-covalent wall association), and KxYKxGKxW (surface display-associated) (Atanasov et al., 2025), which further suggest roles in surface adhesion or host interaction. The putative extracellular proteins with either conserved domains or signal peptides are presented in Table 2.

**TABLE 2:** *In silico*—identified extracellular proteins in *L. cremoris* FBMS_5810, with annotations for domains and signal peptides.

No.	Gene ID	Motif within protein	Signal peptide
1	LCR5810_001239	Class II bacteriocin (Garvicin Q)	No
2	LCR5810_002084	LysM peptidoglycan-binding domain	No
3	LCR5810_002295	WxL domain	Yes
4	LCR5810_002528	LysM peptidoglycan-binding domain	No
5	LCR5810_000790	KxYKxGKxW domain	No
6	LCR5810_001149	–	Yes
7	LCR5810_001461	–	Yes
8	LCR5810_001776	–	Yes
9	LCR5810_002174	–	Yes
10	LCR5810_000489	–	Yes
11	LCR5810_000825	–	Yes
12	LCR5810_000902	–	Yes
13	LCR5810_001793	–	Yes
14	LCR5810_002020	–	Yes
15	LCR5810_002138	–	Yes
16	LCR5810_002363	–	Yes
17-	LCR5810_000113		Yes

### Preclinical study

*In silico* analysis of the *L. cremoris* FBMS_5810 genome revealed numerous cell wall-anchored adhesion proteins, suggesting efficient host surface colonization, and a high number of predicted secreted proteins, indicating potential immunomodulatory effects. Along with the safety assessments, prior *in vitro* studies (Pavlatou et al., 2025) and a small-scale clinical trial (Bousdouni et al., 2025), these findings prompted a preclinical study to systematically evaluate both the safety and functional properties of *L. cremoris* FBMS_5810 in healthy animals.

#### Growth performance and biochemical profile

C57BL/6 mice received a daily oral dose of either 10∧9 CFU of *L. cremoris* FBMS_5810 or 0.2 mL of PBS for seven days, followed by administration every other day for the next 35 days. As shown in Supplementary Figure S1 and Supplementary Table S2, treatment with *L. cremoris* FBMS_5810 did not significantly affect body weight gain, indicating no impact on growth performance. Moreover, no significant differences were observed in any of the biomarkers measured between LC and PBS groups at the end of the intervention (Supplementary Table S3).

#### Histological analysis

Histological analysis revealed intact villus and crypt architecture in both *Lactococcus cremoris* FBMS_5810- and PBS- treated mice after the 6-week intervention (Supplementary Figures S2, S3), with no differences observed between the two groups (Supplementary Figure S2).

#### Analysis of fecal microbiota

To investigate the impact of *Lactococcus cremoris* FBMS_5810 on gut microbiota homeostasis, fecal samples were subjected to 16S rRNA sequencing. A total of 68 bacterial OTUs (at the family level) were initially identified at a 97% sequence similarity. Applying a 0.1% abundance threshold alongside a 25% prevalence rule, the analysis retained 30 bacterial OTUs (Supplementary Tables S4, S5) that met the filtering criteria, while 38 low-abundance OTUs were excluded. The Rarefaction and Shannon curves indicated that the sampling effort was sufficient to encompass the fecal bacterial community structure within the analyzed sample (Supplementary Figures S4, S5).

**FIGURE 5:**
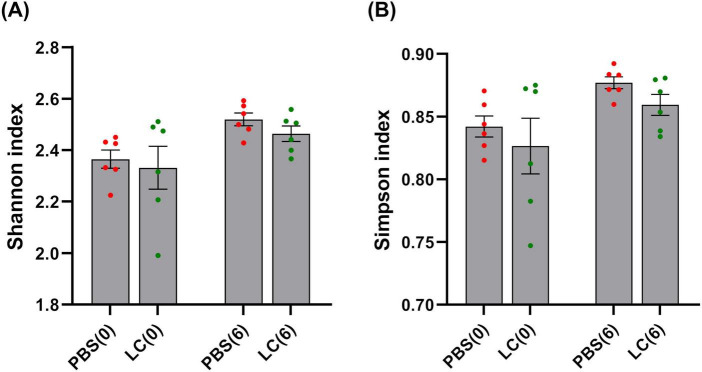
Alpha diversity analysis following *L. cremoris* FBMS_5810 intervention, showing no significant changes in microbial diversity between groups. Alpha diversity is represented by the Shannon **(A)** and the Simpson **(B)** indices. Data are expressed as mean ± SEM. PBS(0), PBS group before the intervention; PBS(6), PBS group after the intervention; LC(0), *L. cremoris* FBMS_5810 group before the intervention; LC(6), *L. cremoris* FBMS_5810 group after the intervention. Differences were not statistically significant (*p* > 0.05).

**FIGURE 6:**
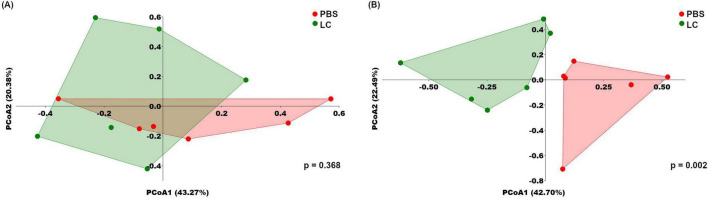
Beta diversity analysis following *L. cremoris* FBMS_5810 intervention, showing a significant shift in microbial community structure after treatment. Beta diversity is visualized by Bray—Curtis distance-based principal coordinates analysis (PCoA) before **(A)** and after the intervention **(B)**. Numbers in brackets indicate the percentage of variance explained by the respective axes (PCoA1 and PCoA2). Statistical significance was assessed using a PERMANOVA test (*p* = 0.002). Each point represents the fecal microbial community of an individual mouse.

Alpha diversity analysis using the two key ecological diversity indices Shannon and Simpson showed that the intervention did not significantly alter microbial richness or diversity (Figure 5). To visualize differences in microbial community structure between groups, Bray–Curtis distance-based PCoA was also conducted (Figure 6). Although the initial microbiota composition appeared similar across groups, a distinct clustering pattern emerged after the 6-weeks intervention, indicating substantial changes in the overall structure of the intestinal microbiota that were independent of the presence and/or absence of rare taxa. The significance of the observed clustering was confirmed using a PERMANOVA test (*p* = 0.002).

**FIGURE 7:**
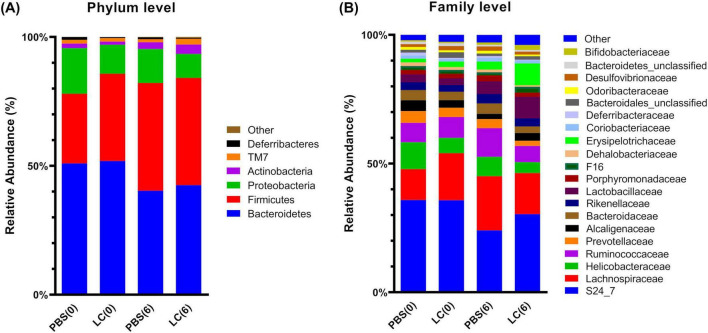
Effect of *L. cremoris* FBMS_5810 on the structure of the fecal microbial community, indicating shifts in the relative abundance of dominant bacterial taxa following intervention. Relative abundance of bacterial OTUs is shown at the phylum **(A)** and family **(B)** levels. OTUs with a median relative abundance below 1% in both groups were grouped as “Other.” PBS(0), PBS group before the intervention; PBS(6), PBS group after the intervention; LC(0), *L. cremoris* FBMS_5810 group before the intervention; LC(6), *L. cremoris* FBMS_5810 group after the intervention.

Next, we analyzed the gut microbiota composition at the taxonomic level. Summary bar plots display the relative abundance of bacterial phyla (Figure 7A) and families (Figure 7B) across groups. Comparisons of the relative abundance of different taxa using Mann–Whitney U-test were executed at both baseline (Supplementary Table S4) and post-intervention (Supplementary Table S5). The predominant phyla across groups included Bacteroidetes, with relative abundances ranging from 35.11 to 60.08%, Firmicutes (ranging from 23.31 to 46.80%), Proteobacteria (ranging from 6.40 to 27.97%), and Actinobacteria (ranging from 0.66 to 6.94%) (Figure 7A). Notably, at baseline, the LC group exhibited a lower relative abundance of the Proteobacteria phylum compared to the PBS group (11.19% vs. 17.51%) (*p* = 0.041, *r* = −0.722, 95% CI for r: −0.920, −0.228) (Figure 7A and Supplementary Table S4). This statistically significant difference was also observed at the end of the intervention (9.10% vs. 12.96%) (*p* = 0.026, *r* = −0.778, 95% CI for r: −0.938, −0.345) (Figure 7A and Supplementary Table S5). No other phyla showed significant differences in relative abundance before or after the intervention.

At the family level after the 6-week intervention, distinct differences between groups (Supplementary Table S5) were observed. The relative abundance of S24_7 (currently known as Muribaculaceae, will be referred to by this name throughout this study) (29.14% vs. 23.31%) (*p* = 0.004, *r* = 0.944, 95% CI for r: 0.800, 0.985), Erysipelotrichaceae (8.12% vs. 3.03%) (*p* = 0.026, *r* = 0.778, 95% CI for r: 0.345, 0.938) and Streptococcaceae (0.69% vs. 0.03%) (*p* = 0.002, *r* = 1, 95% CI for r: 1, 1) was significantly higher in LC group. In addition, the relative abundance of Ruminococcaceae (6.03% vs. 10.81%) (*p* = 0.026, *r* = −0.778, 95% CI for r: −0.938, −0.345), Bacteroidaceae (2.43% vs. 3.87%) (*p* = 0.015, *r* = −0.833, 95% CI for r: −0.954, −0.477), Porhyromonadaceae (1.59% vs. 2.27%) (*p* = 0.041, *r* = −0.722, 95% CI for r: −0.920, −0.228), and Dehalobacteriaceae (0.56% vs. 1.01%) (*p* = 0.048, *r* = −0.694, 95% CI for r: −0.912, −0.175) families was significantly reduced in the *L. cremoris* FBMS_5810-treated group. Remarkably, the family Helicobacteriaceae—a member of the Proteobacteria phylum—was already reduced in the LC group at baseline (5.39% vs. 9.96%) (*p* = 0.041, *r* = −0.722, 95% CI for r: −0.920, −0.228), suggesting that its lower levels observed at the end of the intervention (4.05% vs. 7.30%) (*p* = 0.015, *r* = −0.833, 95% CI for r: −0.954, −0.477), may not be attributable to the probiotic consumption.

#### Gene expression analysis

To investigate the immunomodulatory effects of *L. cremoris* FBMS_5810, we analyzed the mRNA expression profiles of key cytokines (Tgfb, Tnf, Il1b, Il6, Il10), as well as the Toll-like receptor 2 (Tlr2) in intestinal tissues. Additionally, the potential impact of L. *cremoris* FBMS_5810 on gut integrity was assessed by evaluating the mRNA expression of intestinal integrity-regulating genes, including *Zo1*, *Ocln, Jama*, and *Muc2*. Furthermore, the expression levels of genes involved in serotonin (*Sert*) and lipid (*Fiaf, Fitm2*) metabolism were also determined.

**FIGURE 8:**
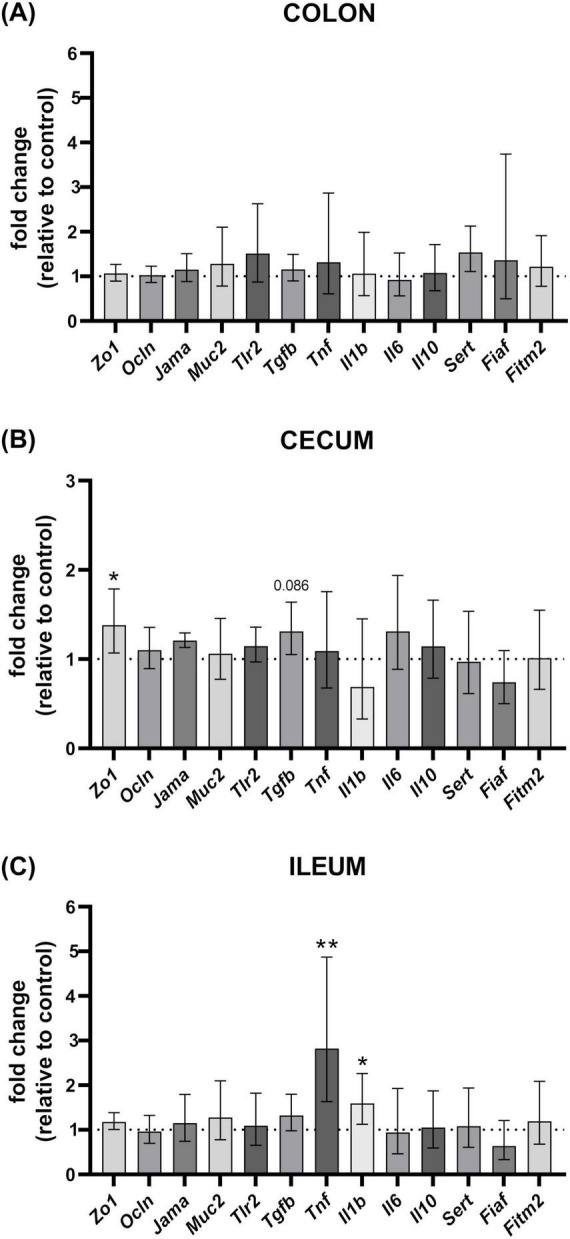
Effect of *L. cremoris* FBMS_5810 on relative gene expression in gut following the 6-week dietary intervention, showing tissue-specific modulation of host markers compared to the PBS control. Relative gene expression in the *L. cremoris* FBMS_5810 treated group was compared with the PBS group in the colon **(A)**, cecum **(B),** and ileum **(C)**. Each bar represents the fold change of the respective marker. Data are expressed as geometric mean with 95% confidence interval. Statistical significance is indicated as follows: **p < 0.01, *p < 0.05, *p* > 0.05 not indicated.

Gene expression analysis in the colon revealed no significant differences between groups (Figure 8A). Regarding the mucosal immune response following *L. cremoris* FBMS_5810 administration, significant alterations were observed in the ileum (Figure 8C), where the LC group exhibited upregulation in the expression levels of *Tnf* (fold change = 2.82) (*p* = 0.007, *d* = −1.940, 95% CI for d: −3.317, −0.501) and Il1b (fold change = 1.59) (*p* = 0.04, *d* = −1.362, 95% CI for d: −2.612, −0.060) compared to PBS group. A tendency to increase was also recorded in the expression of *Tgfb* mRNA in cecum (fold change = 1.31) (*p* = 0.086, *d* = −1.101, 95% CI for d: −2.306, 0.150) (Figure 8B). *L. cremoris* FBMS_5810 administration also led to higher mRNA expression levels of *Zo1* in cecum (fold change = 1.38) (*p* = 0.02, *d* = −1.589, 95% CI for d: −2.885, −0.237) (Figure 8B), indicating a potential enhancement of epithelial barrier integrity.

## Discussion

Following our earlier work, which reported the isolation of *Lactococcus cremoris* FBMS_5810 and several of its probiotic characteristics *in vitro* (Pavlatou et al., 2025), the present work expands this investigation through an integrated genomic and preclinical approach; we examined genomic novelty, phylogenetic placement, and safety profile, while assessing the *in vivo* effects on gut microbiota composition, immune gene expression, and intestinal barrier integrity. Together, our findings support the classification of *L. cremoris* FBMS_5810 as a genetically distinct, safe, and biologically active strain with features consistent with probiotic potential.

The rapidly expanding global probiotic market increasingly requires strains to be thoroughly characterized at the genomic level, to ensure safety, reproducibility and regulatory compliance (EFSA Panel on Additives and Products or Substances used in Animal Feed [FEEDAP], 2018; FAO/WHO, 2002). Our analyses confirmed that FBMS_5810 belongs to the *L. cremoris* species, with ANI values above 95% relative to curated reference genomes, but below the 99% threshold—a meaningful genomic divergence from previously sequenced strains. Comparisons with *L. lactis* yielded ANI values below 90%, indicating its precise taxonomic assignment. Pangenome analysis revealed substantial genomic diversity within the species and identified 157 strain-specific genes not detected in other genomes analyzed, which may contribute to functional specialization, environmental adaptation, or host interaction. The closest phylogenetic relatives of FBMS_5810 were non-dairy isolates, supporting the existence of plant- or non-dairy-adapted ecotypes within *L. cremoris* and their potential application as plant-based probiotics a market segment experiencing growth due to increasing demand for dairy-free functional foods. Regarding safety, two resistance-associated genes were identified: vanY, part of a putative vanG cluster, and lmrD, an ABC-type multidrug efflux transporter. Phenotypic analysis revealed that *L. cremoris* FBMS_5810 is susceptible to vancomycin, indicating that the vanY gene is non-functional, and consistent with previous reports showing that LmrC and LmrD mediate intrinsic, non-transferable resistance (Lubelski et al., 2007). No virulence factors were detected, the strain was classified as “Human Non-Pathogenic” by PathogenFinder2, and hemolytic activity was absent in agreement with the GRAS, QPS, and FOSHU status of most *Lactococcus* species.

Adhesion to host tissues and co-aggregation with other microbes are important probiotic features as they support colonization, persistence, and competitive pathogens exclusion; a key mechanism underlying these processes is the action of sortases and sortase-dependent proteins (SDPs). SDPs, a diverse group of surface proteins, including collagen- and mucin-binding proteins, are covalently anchored to the bacterial cell wall by sortases (van Zyl et al., 2020). Our previous *in vitro* studies showed that *L. cremoris* FBMS_5810 adheres strongly to Caco-2 intestinal cells and exhibits robust co-aggregation with enteric pathogens, including *Clostridioides difficile* (Pavlatou et al., 2025). In line with these findings, genomic analysis identified multiple cell wall– associated proteins with collagen- and mucin-binding domains and LPXTG motifs supporting the strain’s capacity for host interaction and providing the rationale for *in vivo* evaluation.

At baseline, the only difference between PBS and LC groups was a lower relative abundance of Proteobacteria in the latter, driven by reduced Helicobacteriaceae, a major family within this phylum. No other taxa differed significantly, and overall microbial community structure was comparable between the two groups, as indicated by non-significant alpha- and beta-diversity metrics. Following the 6-week intervention, Proteobacteria and Helicobacteriaceae remained lower in the LC group, indicating that *L. cremoris* FBMS_5810 did not measurably affect these taxa. While Shannon and Simpson indices remained unchanged, beta-diversity analysis revealed a clear separation between groups. This pattern is consistent with selective modulation of specific taxa within a stable microbiota, rather than broad changes in overall diversity. Accordingly, *L. cremoris* FBMS_5810 selectively modulated specific microbial taxa, leading to compositional changes that were sufficient to distinguish the groups in the PCoA analysis. Several relatively abundant taxa that were comparable at baseline exhibited significant changes following the intervention. Therefore, although overall diversity remained stable, targeted shifts in key taxa underlie the observed differences in beta diversity, indicating a meaningful yet selective modulation of the gut microbiota.

Notably, the observed microbial shifts suggest modulation of taxa associated with host metabolic and inflammatory status. More specifically, Muribaculaceae, one of the most common families in the mouse fecal microbiota (Lkhagva et al., 2021), displayed a significant increase (by approximately 25%) in relative abundance. Muribaculaceae are recognized as key carbohydrate-degrading bacteria that contribute to short-chain fatty acid (SCFA) production, particularly propionate, which is associated with improved metabolic regulation and anti-inflammatory effects (Zhu et al., 2024). Their increase following *L. cremoris* FBMS_5810 administration therefore suggests an enhanced functional capacity of the microbiota to support intestinal homeostasis. In contrast, although Ruminococcaceae are also involved in fiber degradation, their enrichment has been associated in certain contexts with increased energy harvest and metabolic imbalance (Kaakoush and Morris, 2017; Rajilić-Stojanović et al., 2015; Rodríguez-Daza et al., 2020; Sun et al., 2021). Their reduction in the present study may therefore reflect a shift toward a more balanced metabolic profile. The direction of these microbial changes differs from patterns reported in diet-induced metabolic disorder models, where Muribaculaceae typically decrease and Ruminococcaceae increase (Liu et al., 2019; Rodríguez-Daza et al., 2020). Interestingly, in those studies, the relative abundance of Bacteroidaceae was also increased, whereas it was decreased in the present study. These differences likely reflect the use of healthy animals, suggesting that *L. cremoris* FBMS_5810 may promote or maintain a microbiota configuration associated with metabolic stability rather than reversing an already dysbiotic state. Changes in Erysipelotrichaceae abundance may also be relevant to host immune modulation. Members of this family have been associated with host–microbe interactions affecting immune responses and epithelial function (Veerapandian et al., 2025; Zhou et al., 2024). Their enrichment, together with Muribaculaceae, may contribute to the observed modulation of immune gene expression. Interestingly, similar protective associations involving Muribaculaceae have been reported in intestinal inflammation models (Zhu et al., 2024). A reduced abundance of Muribaculaceae is observed during the development of colitis in dextran sulfate sodium (DSS)-induced mouse models (Huang et al., 2022; Shang et al., 2021). Pre-administration of *Saccharomyces boulardii*-derived postbiotics prevents DSS-induced colitis by maintaining Muribaculaceae levels (Jin et al., 2025), whereas post-DSS treatment ameliorates acute colitis in part, through enrichment of this family (Huang et al., 2022; Zhan et al., 2024). The increase of Muribaculaceae following *L. cremoris* FBMS_5810 administration may indicate a potential anti-inflammatory or gut-protective role, aligning with its effects on microbiota composition.

Beyond their effects on the microbiota, probiotics are recognized for their ability to reinforce the intestinal barrier—both the physical layer of tight junctions between epithelial cells and the mucosal chemical barrier crucial for host defense; disruption of this barrier has been implicated in numerous diseases (Chelakkot et al., 2018). Following *L. cremoris* FBMS_5810 administration in healthy mice, Zo1, a well-established marker of tight junction integrity, was significantly increased in the cecum. Similar results have been observed in broilers supplemented with *C. butyricum* spores (Liu et al., 2022), and in mice, where probiotic administration increased Zo1 expression in colons of both normal and acute colitis models (Wong et al., 2024). As ZO-1 is a peripheral membrane-associated protein essential for maintaining epithelial cohesion (Chelakkot et al., 2018), these findings suggest that *L. cremoris* FBMS_5810 may enhance intestinal barrier integrity even in healthy hosts, potentially supporting gut homeostasis.

Probiotics are also key modulators of the host immune system, with the intestine serving as the body’s largest immune organ (Mazziotta et al., 2023). To assess the immunomodulatory potential of *L. cremoris* FBMS_5810, the expression of a battery of immune-related genes in the colon, cecum, and ileum was analyzed. *L. cremoris* FBMS_5810 administration led to a significant increase in Tnf (2.82-fold) and Il1b (1.59-fold) expression in the ileum, a region characterized by a dense immune cell network and continuous exposure to dietary antigens (Bowcutt et al., 2014). This increase indicates activation of mucosal immune signaling. In the context of a healthy host, this moderate upregulation likely reflects controlled immune stimulation rather than pathological inflammation. Controlled induction of these cytokines by non-pathogenic bacteria is considered a hallmark of probiotic immunomodulatory activity that supports balanced immune activation and maintains intestinal homeostasis (Dogi et al., 2008). Although TNF and IL-1β are traditionally classified as pro-inflammatory mediators, their regulated expression in gut immune cells can enhance host resistance to pathogens and prevent uncontrolled inflammatory responses (Castillo et al., 2011; Pagnini et al., 2010; Salva et al., 2010; Xia et al., 2018). In line with this, Shan et al. proposed that probiotic-induced pre-activation of mucosal immunity may act as a protective mechanism against subsequent pathogenic challenges (Shan et al., 2021). Similarly, monoassociation with *Lactobacillus delbrueckii* UFV-H2b20 in germ-free mice prevented the immunopathological effects of *Listeria monocytogenes* infection by stimulating protective immune responses, such as elevated TNF levels in the serum, peritoneal cavity, and intestinal tissue (dos Santos et al., 2011).

Probiotics also play a crucial role in restoring immune function under immunosuppressive conditions. In mice treated with cyclophosphamide, a broad-spectrum anticancer drug, probiotic supplementation restored Tnf and Il1b expression to physiological levels (Dong et al., 2025; Meng et al., 2019; Zeng et al., 2023). Consistent with these findings, other studies have shown that probiotics enhance immune recovery in immunosuppressed mice through modulation of gut microbiota composition, with cytokines, such as TNF and IL-1β, positively correlating with Muribaculaceae and Erysipelotrichaceae abundance (Zhou et al., 2024). Notably, in immunosuppressed mice—with or without *Candida albicans* infection—depletion of beneficial bacteria from the Muribaculaceae and Erysipelotrichaceae families is accompanied by an overrepresentation of pathogenic taxa, highlighting microbiota dysbiosis as a key contributor to infection severity (Veerapandian et al., 2025). Therefore, the observed upregulation of Tnf and Il1b following *L. cremoris* FBMS_5810 administration may, at least in part, reflect the concurrent increase in Muribaculaceae and Erysipelotrichaceae abundance, suggesting that this microbial shift contributes to the immunostimulatory effects of the probiotic. Beyond immune activation, TNF-mediated signaling is also essential for maintaining colonic epithelial integrity and promoting wound healing. Bradford et al. demonstrated that TNF acts on colonic epithelial stem and progenitor cells, enhancing Wnt/β-catenin signaling and facilitating mucosal repair during colitis (Bradford et al., 2017). Similarly, TNF-induced activation of the growth factor receptor ErbB4 has been shown to support epithelial survival and protect against ulceration in inflamed colonic tissue (Hilliard et al., 2011). These findings collectively indicate that *L. cremoris* FBMS_5810 may promote intestinal health through both immune modulation and epithelial reinforcement. We note, however, that mRNA expression data may not directly reflect protein levels or functional activity; therefore, future studies are needed to confirm these results at the protein and/or functional level. Despite these effects, no significant changes were detected in inflammatory or metabolic serum biomarkers, in contrast to observations from a 12-week randomized, placebo-controlled clinical trial in which *L. cremoris* FBMS_5810 immobilized on oat flakes significantly improved systemic markers of inflammation and metabolism (Bousdouni et al., 2025). This discrepancy may be attributed to differences in intervention duration (12 weeks) or to the use of oat flakes as a delivery matrix, which could influence the survival, colonization, and metabolic activity of *L. cremoris* FBMS_5810 in the gastrointestinal tract.

In conclusion our study provides a comprehensive genomic and preclinical evaluation of *L. cremoris* FBMS_5810, confirming its taxonomic identity, genomic distinctiveness, and lack of safety concerns; genomic analyses revealed strain-specific genes associated with adhesion, colonization, and pathogen exclusion, consistent with previously observed *in vitro* probiotic properties. *In vivo* studies in healthy mice, showed that *L. cremoris* FBMS_5810 induced targeted modulation of gut microbiota, composition and changes the host gene expression, related to immune signaling and barrier integrity, indicating an influence in host–microbiome interactions under physiological conditions. These findings support a role in maintaining intestinal homeostasis; however, as this study was conducted in healthy animals, further investigation in disease-relevant models is required to determine its potential protective or therapeutic effects. Overall, *L. cremoris* FBMS_5810 represents a well-characterized candidate for future probiotic development.

## Data Availability

The Whole Genome Sequencing (WGS) data for *Lactococcus cremoris* FBMS_5810 have been deposited in DDBJ/ENA/GenBank (accession JBSLYB000000000.1). Additionally, the raw 16S rRNA metagenomic sequencing data have been deposited in the Sequence Read Archive (SRA) under the BioProject accession PRJNA1440480.

## Footnotes

1
https://nc3rs.org.uk/3rs-resource-library/blood-sampling/blood-sampling-mouse

2
https://www.gimp.org

3
https://imagej.nih.gov

4
https://www.microbiomeanalyst.ca/

## Supplementary material

Supplementary Figure S1 Body weight analysis during the dietary intervention showing no significant differences between the PBS and *Lactococcus cremoris* FBMS_5810—treated groups. **(A)** Body weight of mice over the course of the intervention and **(B)** total body weight gain % . Data are expressed as mean ± SEM. PBS, PBS group; LC, *L. cremoris* FBMS_5810 group. *p* > 0.05 not indicated.

Supplementary Figure S2 Intestinal morphology following the dietary intervention, showing no evident histological differences between the PBS and *Lactococcus cremoris* FBMS_5810-treated groups. Representative images of colonic **(A,B)**, cecal **(B,C)**, and ileal **(D,E)** tissue sections at the end of the dietary intervention. Scale bars in **(A,B,E,F)** 100 μm, in **(C,D)** 50 μm. PBS, PBS group; LC, *L. cremoris* FBMS_5810 group.

Supplementary Figure S3 Morphometric analysis of intestinal structures following the dietary intervention, showing no significant differences between the PBS and *L. cremoris* FBMS_5810-treated groups. Assessment of colonic **(A)** and cecal **(B)** crypt depth and ileal villus height **(C)** at the end of the dietary intervention. Data are expressed as mean ± SEM. PBS, PBS group; LC, *L. cremoris* FBMS_5810 group. Differences were not statistically significant (*p* > 0.05).

Supplementary Figure S4 Baseline diversity assessment of fecal samples, showing comparable sequencing depth and microbial diversity between groups prior to intervention. Rarefaction **(A)** and Shannon diversity **(B)** curves are shown for each sample, with each line representing an individual sample. PBS, PBS group; LC, L. cremoris_ FBMS 5810 group.

Supplementary Figure S5 Post-intervention diversity assessment of fecal samples, indicating comparable sequencing depth and overall microbial diversity between groups. Rarefaction **(A)** and Shannon diversity **(B)** curves are shown for each sample, with each line representing an individual sample. PBS, PBS group; LC, *Lactococcus cremoris* FBMS_5810 group.
