# Supplementary figures and images for: Genomic characterization and preclinical evaluation of the candidate probiotic strain Lactococcus cremoris FBMS_5810
Source: Front Microbiol. 2026 Apr 29;17:1812433. doi: 10.3389/fmicb.2026.1812433 (PMC13167713; doi:10.3389/fmicb.2026.1812433)

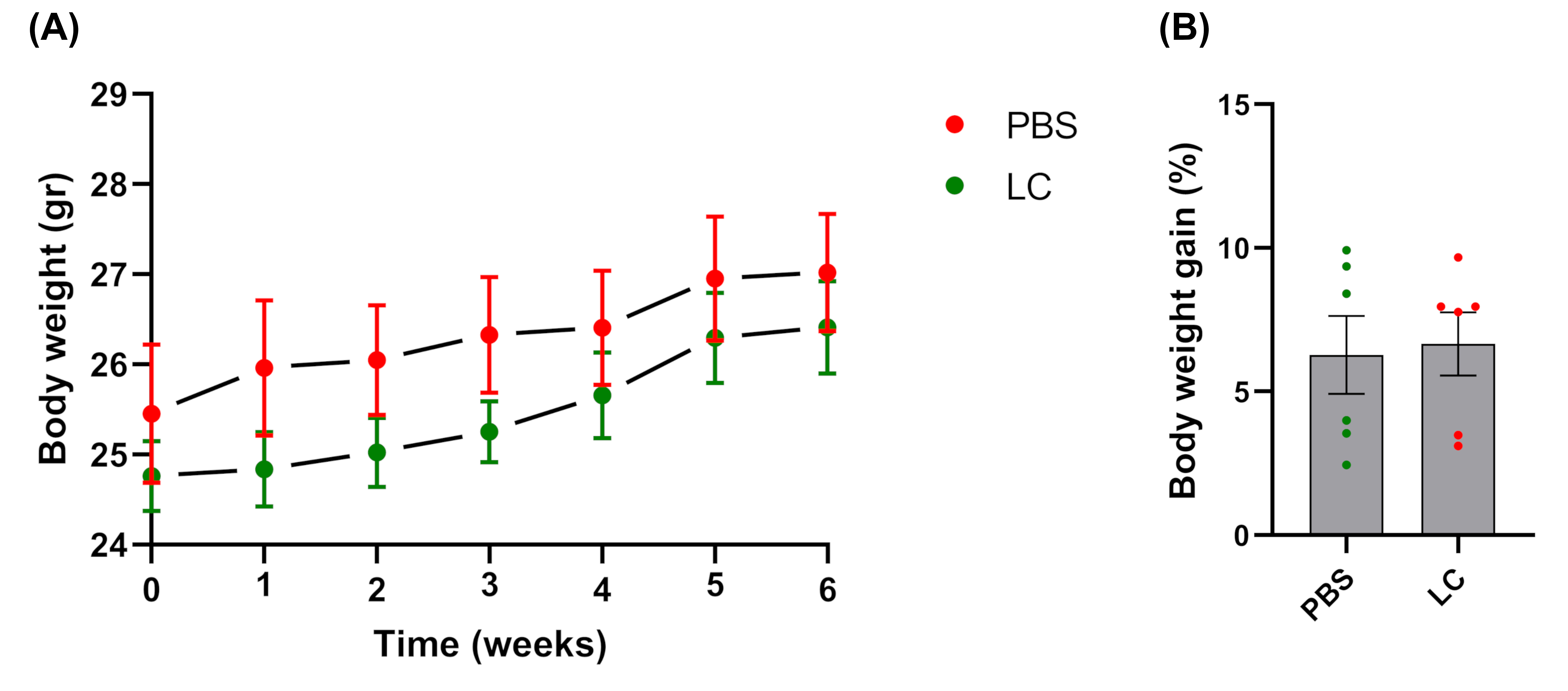

Supplement: Supplementary Figure S1 — Body weight analysis during the dietary intervention showing no significant differences between the PBS and Lactococcus cremoris FBMS_5810—treated groups. (A) Body weight of mice over the course of the intervention and (B) total body weight gain % . Data are expressed as mean ± SEM. PBS, PBS group; LC, L. cremoris FBMS_5810 group. p > 0.05 not indicated. [file Image_1.jpeg]

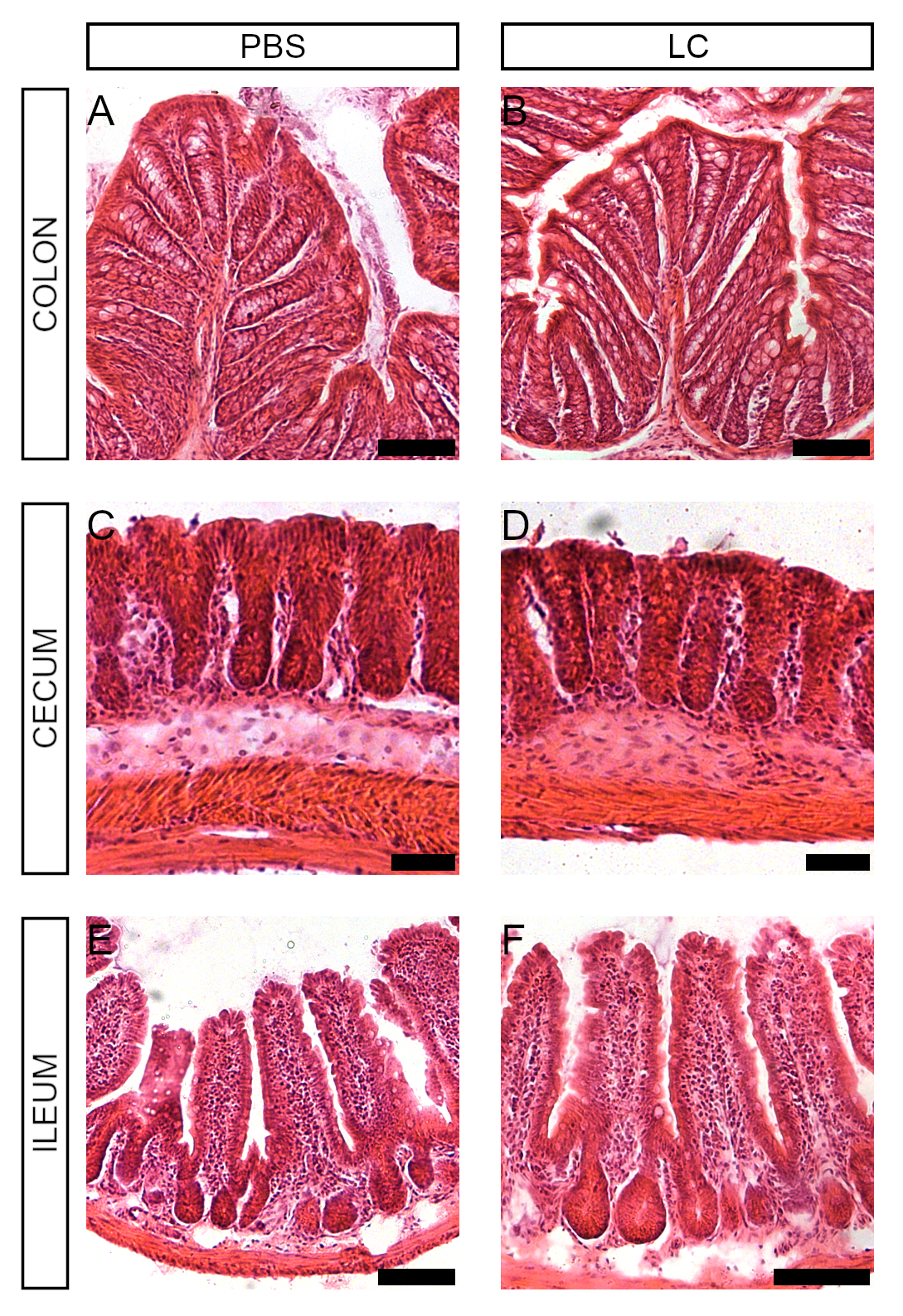

Supplement: Supplementary Figure S2 — Intestinal morphology following the dietary intervention, showing no evident histological differences between the PBS and Lactococcus cremoris FBMS_5810-treated groups. Representative images of colonic (A,B), cecal (B,C), and ileal (D,E) tissue sections at the end of the dietary intervention. Scale bars in (A,B,E,F) 100 μm, in (C,D) 50 μm. PBS, PBS group; LC, L. cremoris FBMS_5810 group. [file Image_2.jpeg]

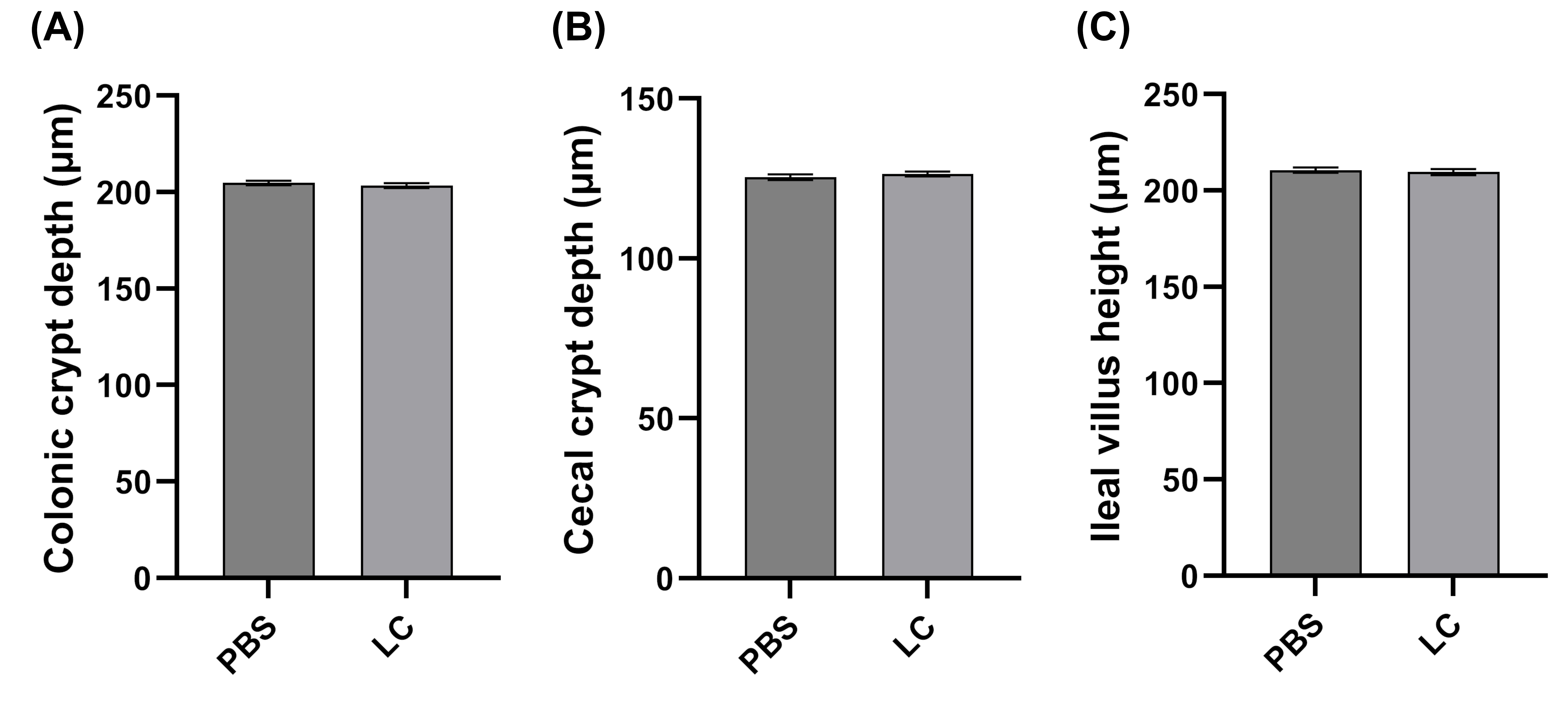

Supplement: Supplementary Figure S3 — Morphometric analysis of intestinal structures following the dietary intervention, showing no significant differences between the PBS and L. cremoris FBMS_5810-treated groups. Assessment of colonic (A) and cecal (B) crypt depth and ileal villus height (C) at the end of the dietary intervention. Data are expressed as mean ± SEM. PBS, PBS group; LC, L. cremoris FBMS_5810 group. Differences were not statistically significant (p > 0.05). [file Image_3.jpeg]

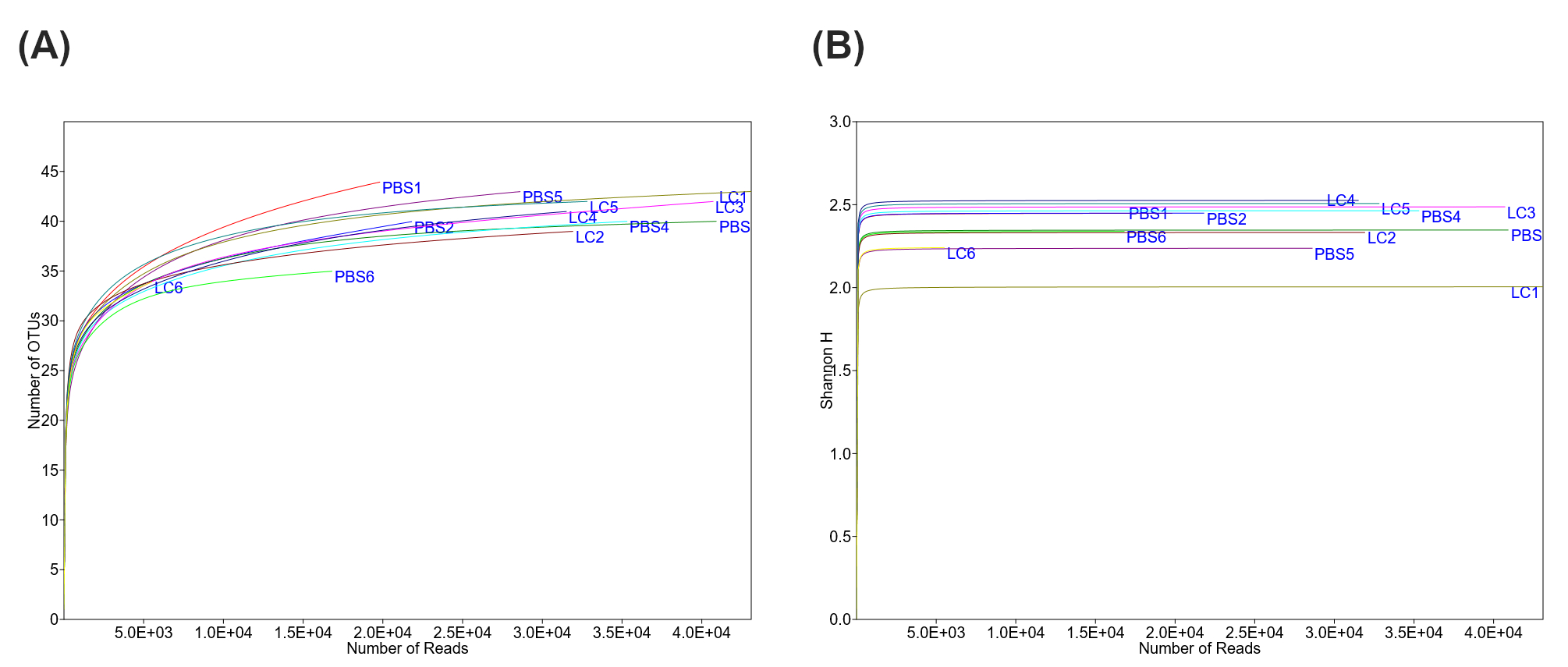

Supplement: Supplementary Figure S4 — Baseline diversity assessment of fecal samples, showing comparable sequencing depth and microbial diversity between groups prior to intervention. Rarefaction (A) and Shannon diversity (B) curves are shown for each sample, with each line representing an individual sample. PBS, PBS group; LC, L. cremoris_ FBMS 5810 group. [file Image_4.jpeg]

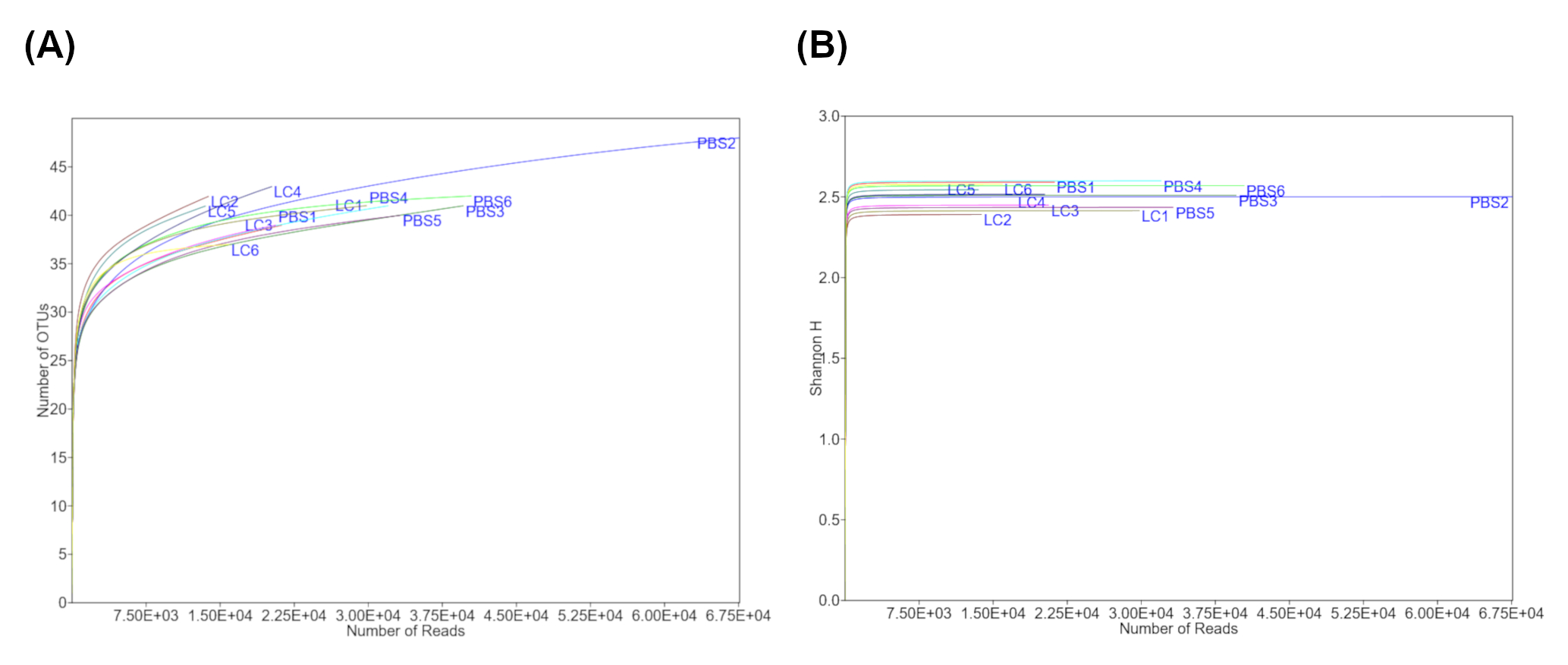

Supplement: Supplementary Figure S5 — Post-intervention diversity assessment of fecal samples, indicating comparable sequencing depth and overall microbial diversity between groups. Rarefaction (A) and Shannon diversity (B) curves are shown for each sample, with each line representing an individual sample. PBS, PBS group; LC, Lactococcus cremoris FBMS_5810 group. [file Image_5.jpeg]
